# Intracellular Redox Perturbation in *Saccharomyces cerevisiae* Improved Furfural Tolerance and Enhanced Cellulosic Bioethanol Production

**DOI:** 10.3389/fbioe.2020.00615

**Published:** 2020-06-23

**Authors:** Chen-Guang Liu, Kai Li, Ke-Yi Li, Chularat Sakdaronnarong, Muhammad Aamer Mehmood, Xin-Qing Zhao, Feng-Wu Bai

**Affiliations:** ^1^State Key Laboratory of Microbial Metabolism, Joint International Research Laboratory of Metabolic and Developmental Sciences, School of Life Sciences and Biotechnology, Shanghai Jiao Tong University, Shanghai, China; ^2^Department of Chemical Engineering, Faculty of Engineering, Mahidol University, Nakhon Pathom, Thailand; ^3^Department of Bioinformatics and Biotechnology, Government College University Faisalabad, Faisalabad, Pakistan

**Keywords:** *Saccharomyces cerevisiae*, redox perturbation, furfural, stress tolerance, ethanol fermentation

## Abstract

Furfural is a major toxic byproduct found in the hydrolysate of lignocellulosic biomass, which adversely interferes with the growth and ethanol fermentation of *Saccharomyces cerevisiae*. The current study was focused on the impact of cofactor availability derived intracellular redox perturbation on furfural tolerance. Here, three strategies were employed in cofactor conversion in *S. cerevisiae*: (1) heterologous expression of NADH dehydrogenase (*NDH*) from *E. coli* which catalyzed the NADH to NAD^+^ and increased the cellular sensitivity to furfural, (2) overexpression of *GLR1, OYE2, ZWF1*, and *IDP1* genes responsible for the interconversion of NADPH and NADP^+^, which enhanced the furfural tolerance, (3) expression of NAD(P)^+^ transhydrogenase (*PNTB*) and NAD^+^ kinase (*POS5*) which showed a little impact on furfural tolerance. Besides, a substantial redistribution of metabolic fluxes was also observed with the expression of cofactor-related genes. These results indicated that NADPH-based intracellular redox perturbation plays a key role in furfural tolerance, which suggested single-gene manipulation as an effective strategy for enhancing tolerance and subsequently achieving higher ethanol titer using lignocellulosic hydrolysate.

## Introduction

Bioethanol is considered as one of the most promising liquid alternatives to fossil fuels, which can be either blended with gasoline or can directly be used as fuel in dedicated engines (Kuhad et al., 2016; Xu and Lin, 2018). The first-generation (1G) ethanol is being produced predominately from starch-based feedstocks. Despite its potential, it cannot be produced from the food-crops-based sugars due to the enormous demands of food supply for the increasing population. Therefore, the production of cellulosic ethanol from lignocellulosic biomass “2G fuel ethanol” has attracted significant attention. However, there are several bottlenecks in the biological transformation of cellulosic biomass to fuel ethanol which includes tedious pretreatments, pretreatment-derived toxic compounds, and inefficient enzymatic hydrolysis. Hence, it is required to develop robust strains to achieve the economic efficiency of 2G fuel production. Along with other problems, the presence of toxic by-products produced during the pretreatment of lignocellulosic biomass is a major feedstock-derived problem, which substantially compromises the growth and fermentation of the yeast (Martín et al., 2018).

Furfural, a representative furan derivative, is present in the biomass hydrolysate (Liu et al., 2004; Wang et al., 2011). It acts as a direct inhibitor of the key enzymes in several pathways, including alcohol dehydrogenase, pyruvate dehydrogenase, acetaldehyde dehydrogenase, etc. (Banerjee et al., 1981), which consequently compromises the normal cell growth and fermentation. Furfural also leads to the accumulation of reactive oxygen species (ROS) in cells (Allen et al., 2010; Benjaphokee et al., 2012; Qiu and Jiang, 2017), which damages cellular components such as lipids, proteins, and DNA. Glutathione and glutaredoxin family are usual antioxidant systems to fight against ROS. NADPH is the only cofactor capable of catalyzing oxidized glutathione (GSSG) to reduced glutathione (GSH) (Almeida et al., 2007). Thus, furfural substantially disturbs the intracellular redox homogeneity either through hindering the synthesis of intracellular reducing power NAD(P)H or accelerating their degradation. It is a common approach to overexpress alcohol or acetaldehyde reductases to enhance *S. cerevisiae* and other industrial microorganisms furfural tolerance because these enzymes promote the conversion of furfural to its less-toxic form, furfuryl alcohol (Moon and Liu, 2012; Hasunuma et al., 2014; Li et al., 2015; Zhong et al., 2016). Besides, some researches focused on improving furfural tolerance by bioprocess engineering or adaptive laboratory evolution. For instance, Wang et al. (2020) applied biochar as a matrix for cell immobilization and as a nutrient supply to improve cell furfural tolerance. Hicks et al. (2020) developed a sequential batch culturing process to increase growth rates and reduced lag time under furfural stress. Previously, we enhanced the furfural tolerance of yeast by extracellular redox regulation under precise air control (Li et al., 2019).

Cofactor engineering to regulate the availability of NADH/NAD^+^ and NADPH/NADP^+^ has demonstrated its role in modulating metabolic networks, signal transduction, material transport, and physiological functions (Liu et al., 2013; Wang et al., 2017a). Therefore, changes in intracellular redox levels propose a theoretical solution to enhance the yeast cell tolerance to furfural. The current study was meant to evaluate the impact of redox perturbation on furfural tolerance. Three types of enzymes were expressed to manipulate the levels of NADH/NAD^+^ and NADPH/NADP^+^ in the cytosol or mitochondria of *S. cerevisiae*. (1), NADH related-genes: *FDH* gene encoding the formate dehydrogenase which catalyzes the NAD^+^ dependent oxidation of formate anion to carbon dioxide (Serov et al., 2002), and *NDH* gene encoding NADH dehydrogenase that catalyzes the transfer of electrons from NADH to the quinone pool in the cytoplasmic membrane of *Escherichia coli* (Salewski et al., 2016). (2), NADPH related-genes: *GLR1* gene encoding cytosolic and mitochondrial glutathione oxidoreductase which reduces the glutathione (Outten and Culotta, 2004), *OYE2* gene encoding NADPH dehydrogenase (Zhao et al., 2017), *ZWF1* gene encoding glucose-6-phosphate dehydrogenase which catalyzes the first step of the pentose phosphate pathway (Cunha et al., 2015), and *IDP1* gene encoding mitochondrial NADP-specific isocitrate dehydrogenase which catalyzes the oxidation of isocitrate to alpha-ketoglutarate (Qin et al., 2015). (3), genes responsible for the interconversion of NADH and NADPH: *POS5* gene localizing in the mitochondria encoding a functional NADH kinase (Shianna et al., 2006), and *PNTB* gene encoding the pyridine nucleotide transhydrogenase of *E. coli* (Clarke et al., 1986). Accordingly, the effects of intracellular redox perturbation caused by cofactor availability on furfural tolerance along with the underlying mechanism, were elucidated.

## Materials and Methods

### Strains and Fermentation

All strains and plasmids used in this study are shown in Table 1. *S. cerevisiae* BY4741 was used as an initial strain to construct the cofactor engineered strains. The *E. coli* DH5α was used for vector construction and propagation. Yeast strains were cultured in 250 mL flasks containing 100 mL YPD medium (yeast extract 10 g/L, peptone 20 g/L, glucose 20 g/L) in a shaking incubator set at 30°C and 150 rpm. In the mid-log phase (around 18 h), cells were inoculated at 10% inoculum into a 2.5 L fermenter containing 1 L fermentation medium (yeast extract 3 g/L, peptone 4 g/L, glucose 100 g/L) supplemented with 4 g/L furfural. The temperature, pH, and rotation rate were set at 30°C, pH 4.5, and 150 rpm, respectively.

**Table 1:** The strains and selected oxidoreductase genes used in this study along with their sources and physiological roles.

**Strain**	**Source**	**Description**	**Function**
BY-FDH*	*S. cerevisiae*	Formate dehydrogenase	formate + NAD^+^ = CO_2_ + NADH
BY-NDH*	*E. coli*	NADH dehydrogenase	NADH + ubiquinone + 5 H^+^ = NAD^+^ + ubiquinol + 4 H^+^
BY-GLR1**	*S. cerevisiae*	Glutathione-disulfide reductase	glutathione disulfide + NADPH + H^+^ = glutathione + NADP^+^
BY-OYE2**	*S. cerevisiae*	NADPH dehydrogenase	NADPH + H^+^ + acceptor = NADP^+^ + reduced acceptor
BY-ZWF1**	*S. cerevisiae*	Glucose-6-phosphate dehydrogenase	D-glucose 6-phosphate + NADP^+^ = D-glucono-1,5-lactone 6-phosphate + NADPH + H^+^
BY-IDP1**	*S. cerevisiae*	Isocitrate dehydrogenase	isocitrate + NADP^+^ = 2-oxoglutarate + CO_2_ + NADPH + H^+^
BY-POS5***	*S. cerevisiae*	NADH kinase	ATP + NAD^+^ = ADP + 2 H^+^ + NADP^+^
BY-PNTB***	*E. coli*	Pyridine nucleotide transhydrogenase	NADH + H^+^ + NADP^+^ = NAD^+^ + NADPH + H^+^

* *NADH related genes*,

** *NADPH related genes*,

*** *NADH to NADPH related genes*.

*E. coli* was cultivated in Luria–Bertani (LB) medium (yeast extract 5 g/L, tryptone 10 g/L, NaCl 10 g/L), a final concentration of 100 μg/L ampicillin was maintained to select the transformants in a 150 mL flask incubated at 37°C, under the constant shaking speed of 200 rpm.

### Genetic Manipulations

DNA sequences of the selected oxidoreductase genes namely, *FDH, GLR1, OYE2, ZWF1, IDP1*, and *POS5* were amplified by polymerase chain reaction (PCR) from the genomic DNA of the BY4741. Whereas, the gene sequences of *NDH* and *PNTB* were amplified from the genomic DNA of *E. coli* DH5α. Primers used in this study are shown in the Table S1. The amplified genes were subcloned into the integration plasmid pHO to obtain overexpression plasmids. Yeast transformation was performed by the LiAc method (Gietz and Schiestl, 2007). The plasmids were transformed into *S. cerevisiae* strains BY4741. Then, the transformants were selected from the YPD agar plates containing 300 μg/mL Geneticin (Sigma-Aldrich, USA).

### Biomass and Metabolites Analyses

A 2 mL of sample was collected from the fermentation broth after every 12 h. Cell growth was measured through optical density at 600 nm (OD600) using a Multiscan Go spectrophotometer (Thermo Scientific, US). After removing cells by centrifugation (10,000 × g for 5 min), the supernatant was subjected to glucose, ethanol, glycerol, and acetate concentrations, and the fermentation broth was analyzed by HPLC system equipped with RI- and UV detectors (Waters e2695, Waters, MA, USA). The Aminex HPX-87H Ion Exclusion Column (Bio-Rad, Hercules, USA) was used to separate the components. Where, 4 mM H_2_SO_4_ was used as a mobile phase at a flow rate of 0.6 mL/min, and the detection temperature of 50°C was used for RI-detector and 65°C for the column. Samples were analyzed in triplicate, and the mean values were calculated.

### Intracellular ROS Content

The sensitive probe 2′,7′-dichlorodihydrofluorescein diacetate (DCFH-DA) was used to measure the intracellular ROS level of cells. Cells were harvested by centrifugation from the 2 mL sample collected after 48 h of the inoculation, washed with phosphate buffer (PBS), and re-suspended in 500 μL PBS supplemented with 40 μL DCFH-DA and incubated at 30°C, 200 rpm for 1 h in darkness. The cells were then collected, washed twice using PBS, re-suspended in 500 μL PBS. From the suspension, a 200 μL mixture was added into each well of a 96-well microplate (black background), and fluorescence intensity was measured by Tecan Infinite 200 microplate reader (Mannedorf, Switzerland) with excitation at λ485 nm and emission at λ535 nm. The relative fluorescence unit was normalized according to the optical density of the cell culture (Allen et al., 2010).

### Intracellular GSH, NADH, and NADPH

NADPH/NADP^+^ and/or NADH/NAD^+^ were estimated based on the role of cofactors in the overexpressed enzymes. The cells (2 mL) were harvested by centrifugation at 48 h after the inoculation, washed twice with PBS and then re-suspended in 1.0 mL of either 0.2 mol/L HCl (for NAD^+^) or 0.2 mol/L NaOH (for NADH) before ultrasonic decomposition. The suspensions were boiled for 5 min, rapidly quenched in an ice bath, and then 500 μL sample was mixed with 500 μL 0.2 mol/L NaOH (for NAD^+^) or 0.2 mol/L HCl (for NADH). Cell debris was removed by centrifugation at 10,000 g for 10 min, and the supernatant was used to determine the amounts of NAD^+^ and NADH following the protocol of the NADH qualification kit (Qiao Suo Co., Ltd., Shanghai, China) as described previously (Wang et al., 2017b). A similar method was used for NAD^+^ and NADH quantification.

GSH and GSSG were detected using GSH and GSSG Assay Kit (Beyotime Biotechnology, Nantong, China) according to the manufacturer's protocol. Cells (2 × 10^5^ /mL) were seeded into 6-well plates and treated with different BQ concentrations (0, 10, and 20 μmol/L) for 24 h. The cells were washed with PBS, and the protein removal agent was added. The samples were frozen and thawed twice with liquid nitrogen in a water bath at 37°C. The cells were centrifuged at 10,000 × g for 10 min at 4°C. The supernatant was used for GSH and GSSG determination. Absorbance was measured at λ450 nm using a microplate reader.

### ATP Measurement

Cells (2 mL) were harvested by centrifugation, washed twice with PBS and then re-suspended in 1 mL cell lysis buffer. From this suspension, 10 μL of the sample was added into 200 μL assay buffer, the mixture was transferred into a 96-well microplate (black background) and fluorescence intensity was measured by Tecan Infinite 200 microplate reader (Mannedorf, Switzerland). The ATP concentration was then calculated according to the ATP standard curve and normalized based on the protein concentration.

### Stress Tolerance Assay

Stress tolerance of yeast cells was evaluated using both plate spot assay and liquid culture assay. For plate spot assay, the strains were cultivated at 30°C in the YPD medium. After overnight culture, OD600 of the broth was adjusted to ~1.0 with distilled water. Ten-fold diluted suspensions were spotted on the YPD plates. Inhibitory compound tested was either acetic acid (4 g/L), furfural (4 g/L), or ethanol (100 g/L). For the heat tolerance test, the plates were incubated at 40°C. The size of the colony was used to evaluate the stress tolerance. For liquid culture assay, strains with initial OD600 of 0.1 were inoculated in 100 mL YPD medium containing 4 g/L furfural in 250 mL flasks. The culturing was performed at 30°C and without pH adjustment. Cell growth (OD600) was measured every 12 h, where higher growth was considered as an indicator of stress tolerance.

### Real-Time Quantitative PCR

Total RNA was isolated from yeast cells and then reversely transcribed into cDNA using a PrimeScript RT Reagent Kit (TaKaRa, Dalian, China) following the instructions of the manufacturer. Real-time quantitative PCR (RT-qPCR) was performed using SYBR Green qPCR Master Mix (TaKaRa, Dalian, China) on a Real-Time PCR Detection System (Bio-Rad, USA). Relative expression levels were calculated by the 2-ΔΔ Ct method (Zhang et al., 2015) using *ALG9* as the reference gene. The sequences of the forward/reverse primers for the analysis were listed in Table S2.

### Heatmap Clustering Analysis and Ternary Plot Analysis

Heatmap clustering analysis was generated using Heml software (http://hemi.biocuckoo.org/down.php), where yields of ethanol, glycerol, and acetate were used as input. Euclidean distance metrics and group clustering were used based on group averages (average linkage). The Ternary plot generated by OriginPro 8 software indicated yields of ethanol, glycerol, and acetate in triangular coordinates with scaled values, which were normalized to restrict the range of values between 0 and 1, for example ethanol yield:

$$\begin{array}{ll} \text{scaled} & \text{ value}=\\ \frac{\log_{10}\left(ethanol\;yield^{*}100\right)}{\begin{array}{c} \log_{10}\left(ethanol\;yield^{*}100\right)+\log_{10}\left(acetate\;yiled^{*}100\right)\\ \;+\log_{10}\left(glycerol\;yield^{*}100\right) \end{array}} \end{array}$$

## Results and Discussion

### Cell Growth of Recombinant Yeast

**Figure 1:**
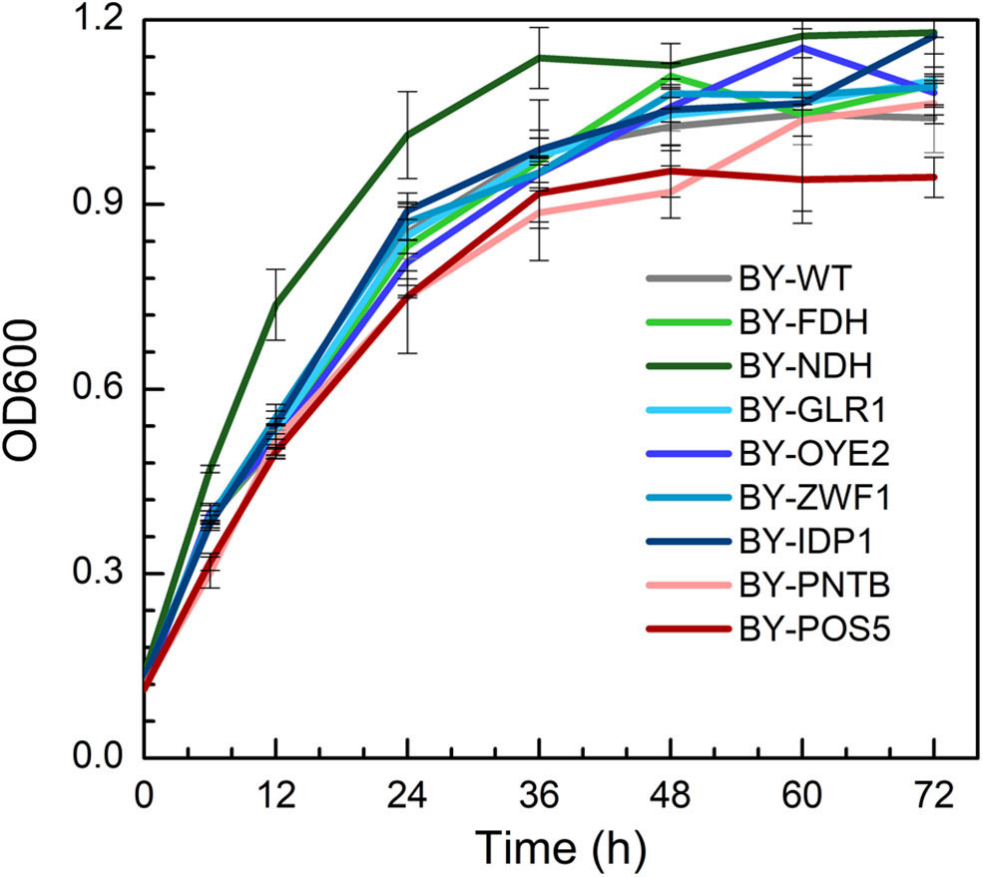
The growth profiles of wild type (BY4741) and engineered strains expressing selected oxidoreductase genes.

The growth of engineered strains was compared with wild type in the YPD medium containing no furfural (Figure 1). It was found that the strain expressing NADH dehydrogenase (BY-NDH) had the best cell growth when compared to wild type and other mutants because the NADH dehydrogenase might have enhanced the respiratory flux of the strain. Conversely, the expression of both *POS5* and *PNTB* had a little negative impact on the growth of the strains. As an NADH kinase, POS5 transforms NADH to NADPH by consuming ATP, which would have constrained the growth due to a lack of enough energy supply. However, as a pyridine nucleotide transhydrogenase, PNTB directly transforms NADH to NADPH without ATP consumption. Thus, intracellular imbalance of NADH and NADPH might have resulted in undesirable yeast growth. The other strains overexpressing the *FDH, GLR1, OYE2, ZWF1*, and *IDP1* genes did not show any influence on cell growth when compared to growth profiles of the wild type.

The yields of ethanol, glycerol, and acetic acid are shown in Table S3. The expression of NADH kinase (*POS5*) or pyridine nucleotide transhydrogenase (*PNTB*) increased the glycerol yield but decreased the yield of acetic acid. Whereas, the expression of NADH dehydrogenase (*NDH*) decreased glycerol yield. On the other hand, BY-ZWF1 and BY-GLR1 showed higher ethanol and glycerol yields when compared to the wild type. It might have provided excessive GSH, which helped the cells to build a more reduceable intracellular environment.

### Tolerance of Recombinant Yeast With NADH/NAD^+^ Ratio Change

**Figure 2:**
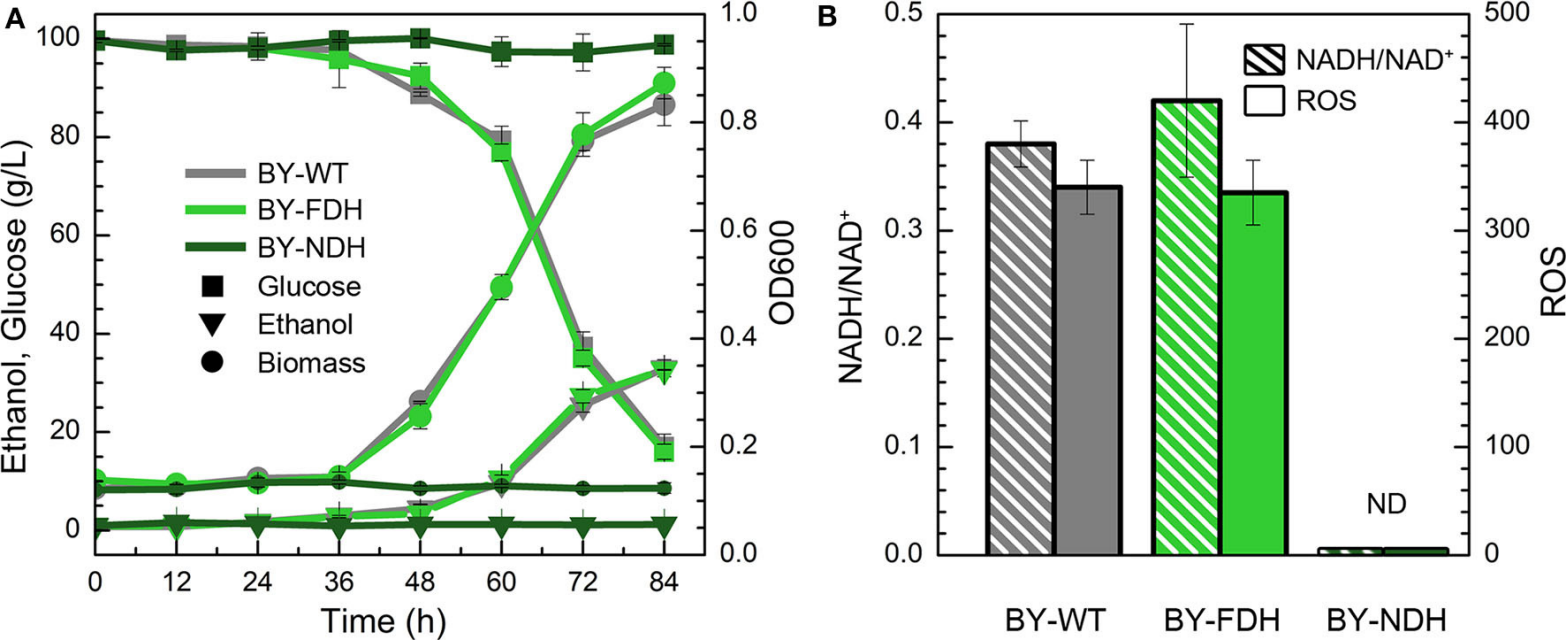
Effect of NADH-related genes expression on yeast cell under the furfural stress (4 g/L). **(A)** Biomass, ethanol, and residual glucose; **(B)** Ratio of NADH/NAD^+^ and ROS content. ND, not detectable.

In the YPD medium containing furfural (4 g/L), the wild type strain BY showed a lag phase spanning over 36 h (Figure 2A), whereas the engineered strain BY-NDH was unable to grow although it had a good performance in the inhibitor-free medium (Figure 1) and it neither consumed any sugar nor produced ethanol under furfural stress. However, the strain BY-FDH, which was overexpressing formate dehydrogenase, exhibited a similar growth curve as that of the wild type strain in the presence of furfural. The glucose consumption of the wild type BY strain and the engineered BY-FDH strain showed a rapid decline after 36 h leaving 37 g/L residual glucose after 72 h (Figure 2A). This was consistent with the previous results that there is no noticeable difference in FDH strain without formate addition (Hou et al., 2010). Formate is a necessary substrate for formate dehydrogenase to produce NADH. However, because formate acts as a toxic carboxylic acid, and it would have interfered with the assessment of furfural tolerance, it was not supplemented into the medium in this study.

To further explain the phenomenon, the ratios of NADH/NAD^+^ and ROS content were measured in BY and BY-FDH. Unfortunately, BY-NDH did not grow under furfural stress, and resultantly produced too little biomass to perform the subsequent analyses. Figure 2B showed there was no substantial difference in NADH/NAD^+^ between wild type and BY-FDH. As discussed above, without extra formate addition, the yeast endogenous formate was too little to maintain the supply required for FDH to accumulate excessive NADH.

ROS are often appeared in universal stress response (Allen et al., 2010). Thus, intracellular ROS content might be an indicator of furfural stress. In consideration of the same performance of growth, ethanol, glucose, and the ratio of NADH/NAD^+^, it was not surprising to see that ROS in BY and BY-FDH had no significant differences. Generally, cell tolerance to furfural can be enhanced by increasing the intracellular NADH ratio, but in this study, it was observed that two engineered strain BY-FDH and BY-NDH did not improve the yeast tolerance to furfural due to shortage of formate and showed significantly poor growth.

### Tolerance of Recombinant Yeast With NADPH/NADP^+^ Ratio Change

**Figure 3:**
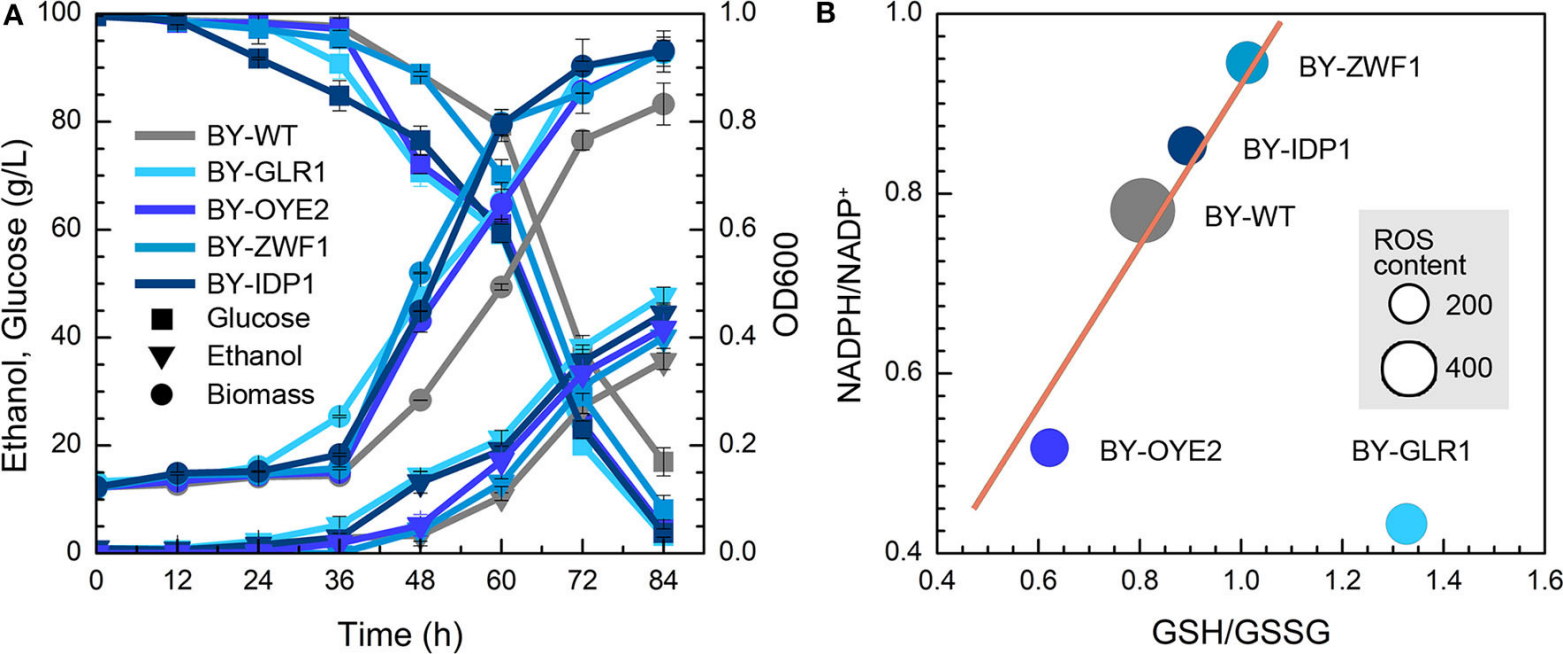
Effect of NADPH-related genes expression on yeast cell under the furfural stress (4 g/L). **(A)** Biomass, ethanol, and residual glucose; **(B)** Content of ROS and ratio of NADPH/NADP^+^ and GSH/GSSG. The size of circles reflects the ROS fluorescence intensity.

The impact of NADPH availability in furfural tolerance was further investigated due to no improvement in strains after overexpression of NADH-related genes. However, both BY-GLR1 and BY-OYE2 showed better growth compared to the wild type in the presence of furfural (Figure 3A). BY-GLR1 even shortened the lag period to 12 h when compared to the wild type. Although BY-OYE2 had the same lag phase with BY, it showed better growth after 36 h. Moreover, BY-OYE2 and BY-GLR1 consumed glucose much quickly, leaving the lower content of residual glucose after 72 h than wild type. Correspondingly, the ethanol production curves of BY-GLR1 and BY-OYE2 were similar and better than the control.

Both strains BY-GLR1 and BY-OYE2 maintained a lower ratio of NADPH/NADP^+^, which was about 80 and 51% of the wild type strain BY (Figure 3B) because the enzymes encoded by *GLR1* and *OYE2* genes consumed NADPH in cells. The glutathione reductase (GLR1) converts oxidized glutathione to reduced glutathione by using NADPH. An NADPH oxidoreductase called old yellow enzyme (OYE2) catalyzes geraniol into citronellol by consuming NADPH (Zhao et al., 2017).

Figure 3B combined the data of NADPH/NADP+, GSH/GSSG, and ROS in recombinant strains and showed their correlation. The linear relationship between NADPH/NADP^+^ and GSH/GSSG can be found in strains BY-ZWF1, BY-IDP1, BY-OYE2, and BY-WT, which reflected that an increase of NADPH caused the accumulation of reduced form of GSH. On the other hand, both BY-IDP1 and BY-ZWF1 had higher ratios of NADPH/NADP^+^ because they encoded glucose-6-phosphate dehydrogenase and isocitrate dehydrogenase. Furthermore, intracellular accumulation of NADPH was also beneficial for the intracellular regeneration of GSH. That is why these two strains (BY-IDP1 and BY-ZWF1) showed higher GSH/GSSG ratios when compared to the wild type and BY-OYE2. However, GLR1 encoded the glutathione reductase, which reduced glutathione by consuming NADPH. Accordingly, the highest ratio of GSH/GSSG was observed in the strain overexpressing *GLR1*, which was a unique plot due to its reaction. Moreover, it was interesting to see that the wild type strain had the highest ROS content when compared to any of the four recombinant strains, which strengthen the hypothesis that more intracellular NADPH produced more intracellular GSH, which conferred a better tolerance to furfural. Interestingly, in previous studies, the extracellular ORP regulation had also enhanced cell growth in the presence of furfural. Cells under ORP regulation had shown a faster GSH generation when compared to the cells with no ORP regulated condition (Li et al., 2019). These results are in accordance with another study, which was meant to improving yeast oxidative stress tolerance by adding redox reagent in the medium (Li et al., 2020). So, both extracellular and intracellular redox perturbation can improve furfural tolerance by increasing intracellular GSH content. To increase intracellular GSH content, more NADPH is needed.

### Tolerance of Recombinant Yeast With NADH-NADPH Transformation

Since NADPH manipulation had a positive effect on yeast tolerance to furfural, the NAD^+^ transhydrogenase and NAD^+^ kinase were overexpressed in the BY to increase the NADPH pool. The NADPH-NADP^+^ and NADH-NAD^+^ systems are separated in yeasts due to the absence of enzymatically catalyzed pyridine nucleotide transhydrogenation and NAD(H) kinase activity (Anderlund et al., 1999). The lack of pyridine nucleotide transhydrogenation has shown considerable consequences for the redox balances of the NAD(H) and NADP(H) coenzyme systems in yeasts (Dijken and Scheffers, 1986). NADH can be transformed to NADPH by the transhydrogenase (PNTB) or NADH kinase (POS5).

**Figure 4:**
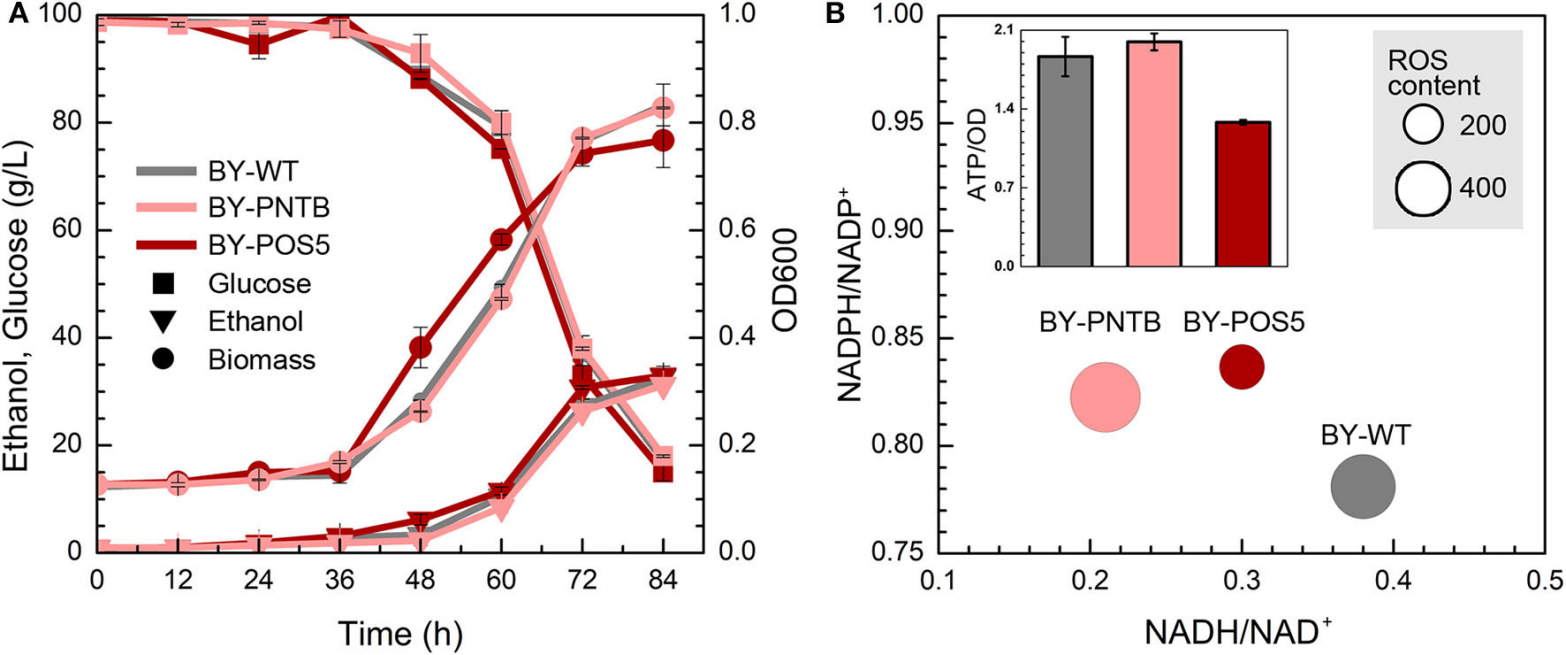
Effect of NADP^+^ transhydrogenase (*PNTB*) and NAD^+^ kinase (*POS5*) expression on yeast cell under the furfural stress (4 g/L). **(A)** Biomass, ethanol, and residual glucose; **(B)** Content of ROS and ATP, and ratio of NADPH/NADP^+^ and NADH/NAD^+^; The size of circles reflects the ROS fluorescence intensity.

Although the expression of both *PNTB* and/or *POS5* genes weaken the cell growth in the YPD medium (Figure 1). Both engineered strains showed similar growth like the wild type in the presence of furfural (Figure 4A), of which BY-POS5 had a slightly better performance. It has been reported that POS5 enzyme is required as a cellular factor for protection from oxidative stress in *S. cerevisiae* (Outten et al., 2005). Ratios of NADH/NAD^+^, NADPH/NADP^+^, and ROS content are shown in Figure 4B. Since PNTB and POS5 catalyzed NADPH formation via consuming NADH; these two enzymes increased the NADPH availability at the cost of decreasing intracellular NADH. Besides, ROS content was significantly decreased in the strain overexpressing the *POS5* gene, which was consistent with cell growth (Figure 4A). However, the ROS content of the strain overexpressing the *PNTB* gene showed almost no difference with the ROS content of wild type strain. The ATP content further demonstrated that *POS5* expression involved the consumption of ATP in NADH to NADPH conversion. *PNTB* expression did not influence the intracellular ATP content (Figure 4B). These phenomena confirmed the function of these two genes.

**Figure 5:**
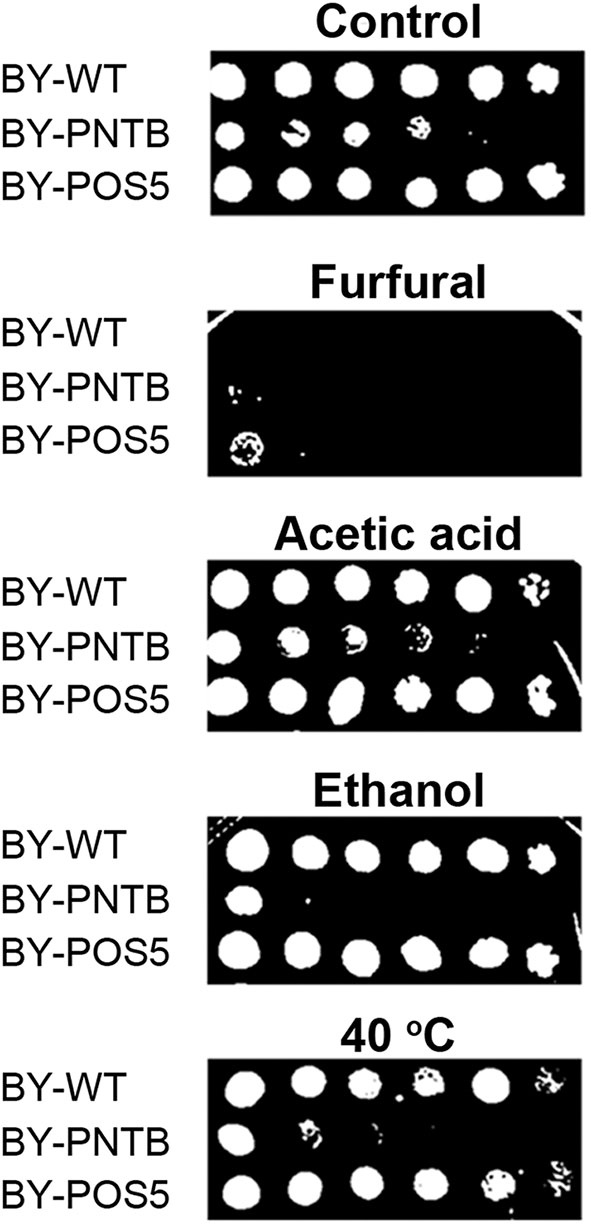
Tolerance of strains expressing either *POS5* or *PNTB* by plate spot assay. Stress factors were acetic acid (4 g/L), furfural (4 g/L), ethanol (100 g/L), and temperature 40°C.

Since POS5 and PNTB could have an impact on cell growth and furfural tolerance, and it was further detected if these two genes have an impact on other stress tolerance. It was found that the expression of the *PNTB* gene defected the cell growth in both solid and liquid YPD medium, and the expression of the *POS5* gene defected the cell growth in the liquid YPD medium but showed no significant difference in the solid medium when compared to the wild type (Figure 5). However, both strains enhanced tolerance against furfural, which was consistent with the cells in the liquid medium. Unfortunately, these two genes did not confer tolerance against any other stresses. Especially the expression of the *PNTB* gene adversely affected the cell growth under ethanol, acetic acid, or heat (40°C) stress. A possible reason is that NADPH is more important than NADH for furfural stress but not for other stresses.

### Relative Transcription Levels of Genes in Engineered Strains

**Figure 6:**
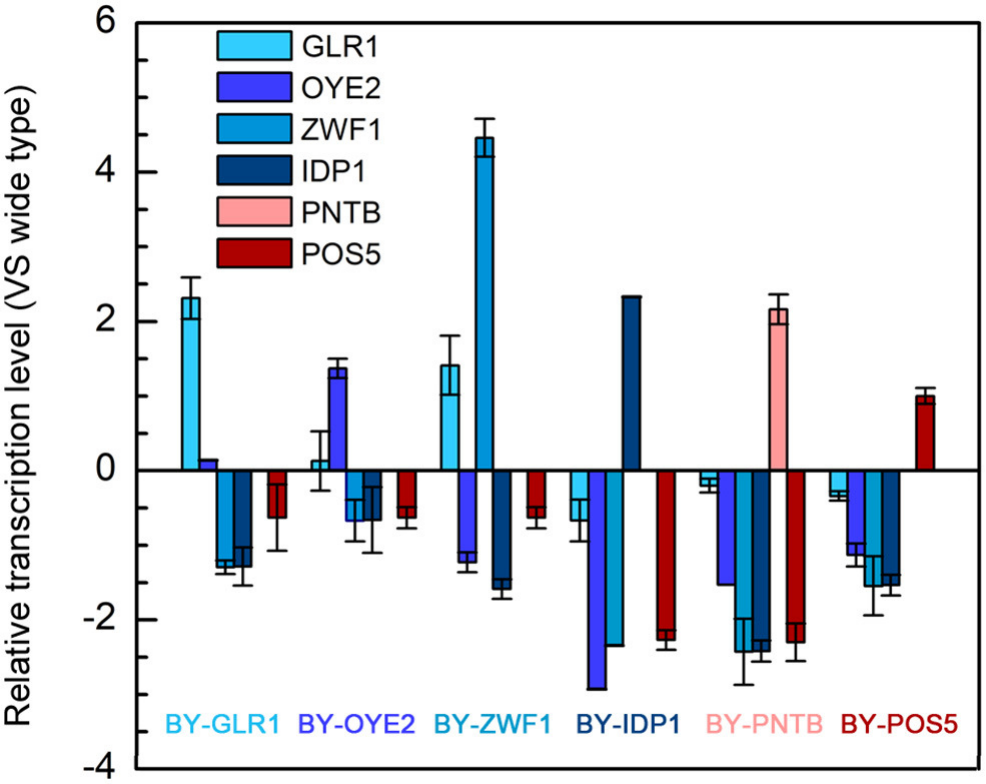
Relative transcription levels of genes in six engineered strains each overexpressing one of the genes including *POS5, OYE2, GLR1, PNTB, ZWF1*, and *IDP1*.

As discussed in section Tolerance of Recombinant Yeast With NADPH/NADP^+^ Ratio Change and Tolerance of Recombinant Yeast With NADH-NADPH Transformation, since the NADPH-related genes enhanced the furfural tolerance in yeast, the transcription level of these genes, including *POS5, PNTB, GLR1, OYE2, ZWF1*, and *IDP1* were analyzed (Figure 6). At first, the successful expression of these genes was confirmed because almost every gene in the recombinant strain was up-regulated individually. Moreover, it was interesting to see that overexpression of any gene led to significant depression of the other NADPH-related genes. However, the transcription level of the *GLR1* gene was shown to be significantly enhanced in the strain overexpressing *ZWF1* gene, possibly due to the reason that glutathione oxidoreductase encoded by *GLR1* would have reduced glutathione by using NADPH while glucose-6-phosphate dehydrogenase encoded by ZWF1 is the main source of NADPH generation. Similarly, the *ZWF1* and *GLR1* were shown to be upregulated under furfural and HMF stress (Liu and Ma, 2020). This result indicated that all genes related to NADPH might own a tight connection; thus manipulation of one gene can change the expression of multiple genes.

### Effect of Cofactor Perturbation on Metabolic Flux

**Figure 7:**
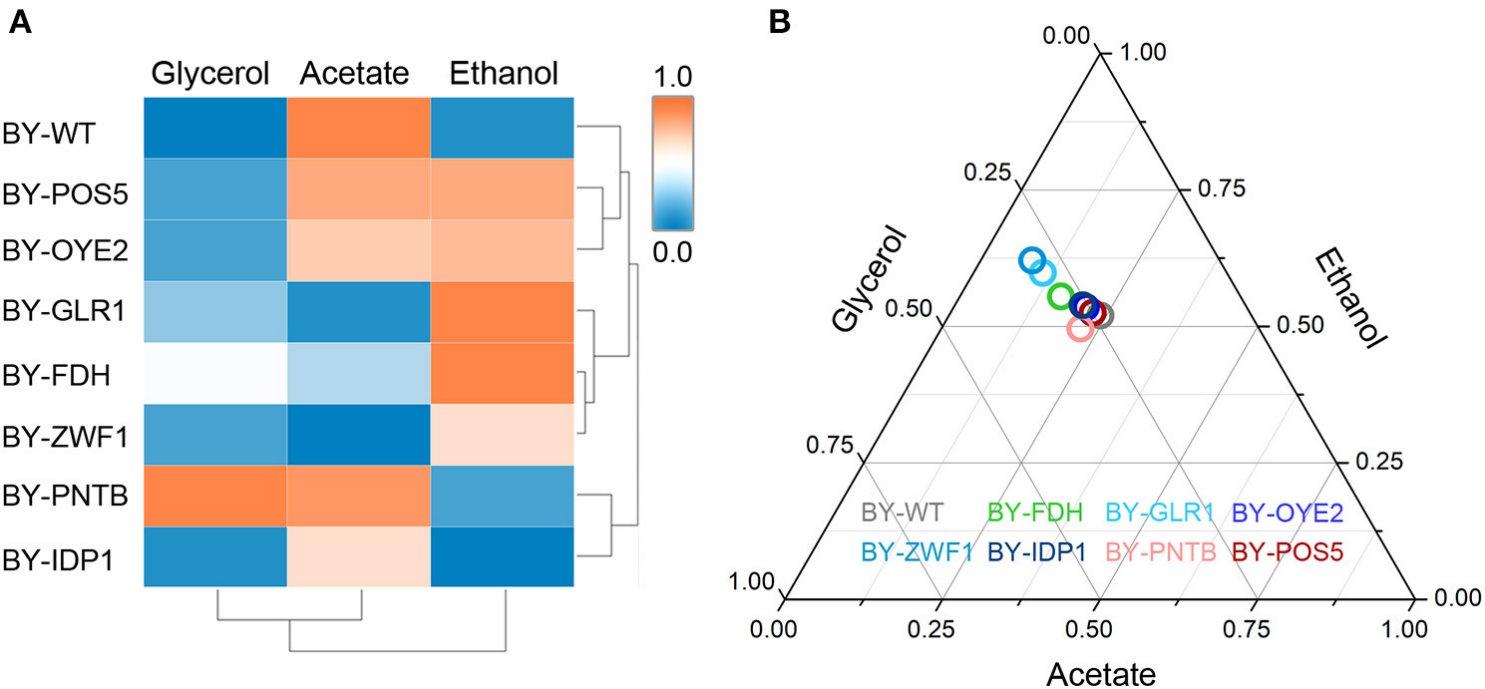
Influence of cofactor-related genes expression on the yield of main products in yeast. **(A)** Heatmap for cluster analysis; **(B)** Ternary plot analysis. All data have been normalized to the range between 0 and 1.

The importance of redox perturbation was further studied for ethanol production by analyzing metabolic flux. A relationship was established among the yields of ethanol, glycerol, and acetic acid in the presence of furfural (Figure 7). The wild type BY strain showed both the lowest ethanol and glycerol yields but had a higher oxidized product yield (acetic acid). However, with the overexpression of glutathione oxidoreductase (GLR1), the highest ethanol yield and the lowest acetic acid yields were observed due to excessive GSH, which produced a more reduceable cellular environment. At the same time, the strains overexpressing *IDP1* or *ZWF1* genes showed a lower acetic acid yield. Similarly, the yields of acetic acid, ethanol, or glycerol showed antagonistic relationships under cofactor disturbed conditions (Bloem et al., 2016). Interestingly, although BY-PNTB showed negative growth in normal medium, yet it had the highest yields of glycerol. In a previous study, the *PNTB* expression had also increased glycerol and acetate content in *S. cerevisiae* (Anderlund et al., 1999).

The cluster analysis showed that BY-GLR1 and BY-ZWF1 belonged to one group though they catalyze different reactions because GLR1 consumes NADPH, but ZWF1 produces NADPH. Additionally, bubbles representing the strains overexpressing *ZWF1* and *GLR1* strains also showed a close relationship when compared to the other strains, which was also seen in the Ternary plot, which was based on the yields of ethanol, acetate, and glycerol (Figure 7). These two strains exhibited a higher ethanol yield but lower glycerol and acetate yield. Both strains are related to GSH synthesis. *GLR1* encoded glutathione oxidoreductase, converted oxidized glutathione to reduced glutathione directly. *ZWF1* encoded glucose-6-phosphate dehydrogenase, catalyzed the first step of the pentose phosphate pathway, which was the main source of intracellular NADPH and indirectly enhanced GSH production. Therefore, these two strains showed an increasing ethanol yield when compared to the other strains in the presence of furfural.

### Mechanism of Redox Perturbation During Furfural Tolerance

**Figure 8:**
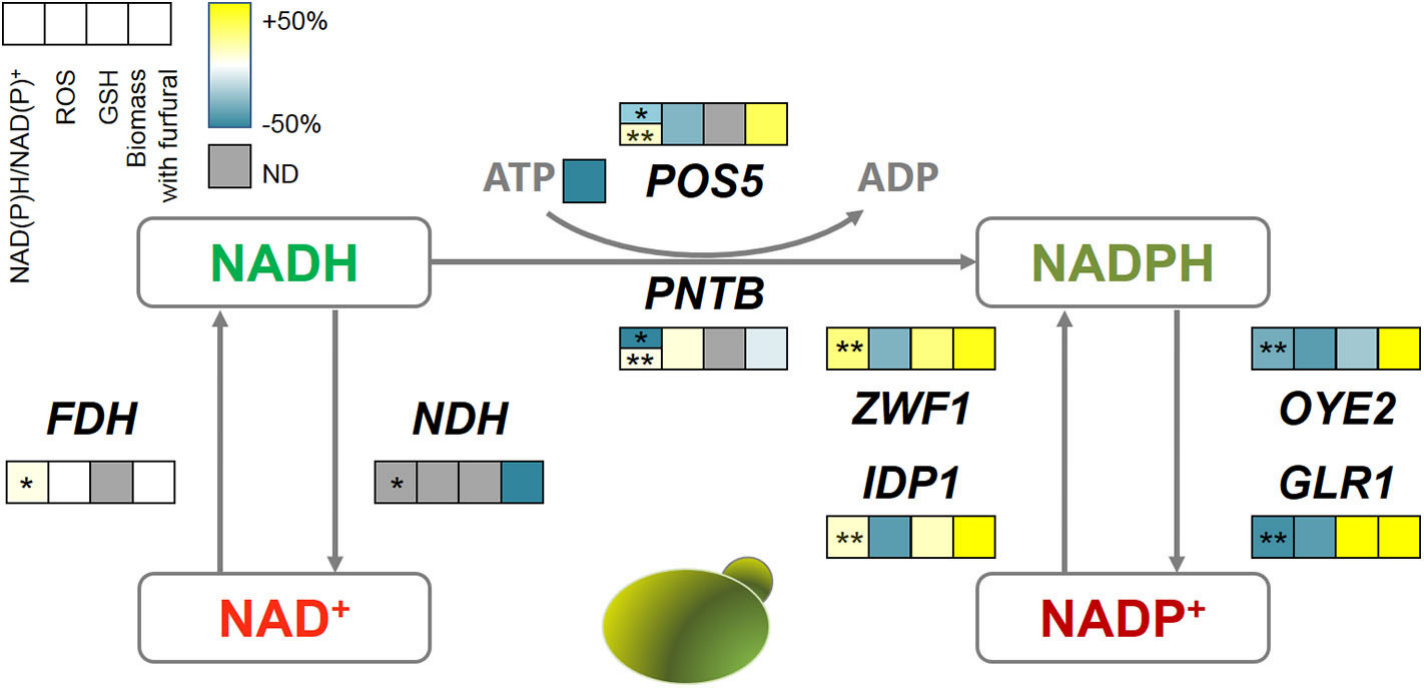
The correlations among oxidoreductases, intracellular redox perturbation, and furfural tolerance. The color bar reflects the improvement defined as (GM-WT)/WT, GM, gene-modified strain; WT, wild type strain. The first square with *shows the NADH/NAD^+^ and with **shows NADPH/NADP^+^. ND, not detectable.

To elucidate the role of intracellular redox perturbation in conferring the furfural tolerance to yeast, the data of redox change, ROS removal, GSH generation, and biomass production under furfural stress, were compared (Figure 8). It clearly showed that the manipulation of oxidoreductases is capable of causing redox perturbation according to their defined bioreaction (Table 1), except for FDH and NDH. Formate dehydrogenase (FDH) produces NADH by consuming formate, which is a trace chemical normally involved in the yeast metabolism (Puig-Castellví et al., 2015). But formate as a carboxylic acid is also a kind of inhibitor that is not supplemented into the medium for evaluation of furfural tolerance to avoid false results. Thus, this enzyme did not substantially contribute to the transformation of NADH, which consequently led to the unchanged ROS accumulation and cell growth under furfural stress. The strain overexpressing NDH did not survive when exposed to 4 g/L furfural, but the details of NADH/NAD^+^ and ROS are not available as well due to the unavailability of the biomass for the required analyses.

Interestingly, almost all genes related to NADPH metabolism benefited furfural tolerance, though some of them have contradictory reaction directions. Because POS5, ZWF1, and IDP1 acted as producers of NADPH, which assisted the anti-oxidative system such as GSH in removing ROS, and thus increased the biomass under furfural stress. Despite causing the depletion of NADPH, the NADPH dehydrogenases (encoded by *OYE2* and *GLR1*) helped the cells to manage a lower ROS content. *OYE2* gene encodes old yellow enzyme that is believed to be involved in sterol metabolism, oxidative stress response, and programmed cell death (Odat et al., 2007). *GLR1* encodes glutathione reductase that directly converts oxidized glutathione (GSSG) to reduced glutathione (GSH) for ROS removal (Heer et al., 2009). It should be noted that PNTB and NDH were heterologously expressed from *E. coli* in yeast. Where, NDH causes cell death under 4 g/L furfural, and PNTB neither influenced the ROS nor the furfural tolerance.

In this study, the improvement of the furfural tolerance of yeast by the redox perturbation through expressing NADH-related genes was not successful. But it did not mean that the change of NADH is invalid for improving furfural tolerance (Wang et al., 2017b). Whereas, the redox perturbation caused by NADPH-related genes has shown promising improvement in furfural tolerance.

## Conclusion

In this study, the importance of cofactor in furfural tolerance by expressing genes related to NADH or NADPH was examined. Where, it was shown that after the heterologous expression of *NDH* gene (an NADH related gene), the strain became extremely sensitive to furfural and was unable grow in medium containing 4 g/L of furfural, while the strain overexpressing *FDH* gene had similar growth when compared to the wild type due to lack of formate as a substrate. The overexpression of NADPH related genes, all five engineered strains, namely BY-POS5, BY-ZWF1, BY-IDP1, BY-GLR1, and BY-OYE2, showed better performance under furfural stress when compared to the wild type strain. This indicated that NADPH is more critical to improve yeast furfural tolerance than NADH because of its role in regenerating antioxidant, which can clear the ROS produced under furfural stress. But here, a different way was followed to encounter the ROS. The enzymes encoded by *POS5, ZWF1, IDP1* replenished the supply of reducing power NADPH, but *GLR1* and *OYE2* directly eliminated ROS by using NADPH. Moreover, the metabolic flux analysis revealed that cofactor perturbation switched the metabolic flux to improve fermentation performance. Furfural induced the accumulation of ROS in the cell, and effective redox perturbation cleared up the ROS, which helped the cells to cope with the furfural stress. This study provided a novel perspective to improve the cellular tolerance through intracellular redox perturbations by a single-gene manipulation.

## Data Availability Statement

The raw data supporting the conclusions of this article will be made available by the authors, without undue reservation.

## Author Contributions

KL and C-GL conceived the study, wrote the manuscript, and prepared figures and tables. KL and K-YL performed experiments and data analysis. X-QZ, MM, CS, and F-WB discussed and revised the manuscript. All authors read and approved the final manuscript.

## Conflict of Interest

The authors declare that the research was conducted in the absence of any commercial or financial relationships that could be construed as a potential conflict of interest.
