# Supplementary material for: Intracellular Redox Perturbation in Saccharomyces cerevisiae Improved Furfural Tolerance and Enhanced Cellulosic Bioethanol Production
Source: Front Bioeng Biotechnol. 2020 Jun 23;8:615. doi: 10.3389/fbioe.2020.00615 (PMC7324476; doi:10.3389/fbioe.2020.00615)
Supplement: Supplementary file 1 [file Data_Sheet_1.pdf]

Supplementary information

Table S1. Primers used in this study

Genes		Primer sequences
FDH	F:	TTTACAACAAATATAAAACAATGAAGATCGTTTTAGTCTT
	R:	AAATTCGCTTATTTAGAAGTTTATTTCTTATCGTGTTTAC
NDH	F:	TTTACAACAAATATAAAACATTGACTACGCCATTGAAAAA
	R:	TGACATAACTAATTACATGATTAATGCAACTTCAAACGCG
GLR1	F:	TTTACAACAAATATAAAACAATGCTTTCTGCAACCAAACA
	R:	TGACATAACTAATTACATGATCATCTCATAGTAACCAATT
OYE2	F:	TTTACAACAAATATAAAACAATGCCATTTGTTAAGGACTT
	R:	TGACATAACTAATTACATGATTAATTTTTGTCCCAACCGA
IDP1	F:	TTTACAACAAATATAAAACAATGAGTATGTTATCTAGAAG
	R:	TGACATAACTAATTACATGATTACTCGATCGACTTGATTT
ZWF1	F:	TTTACAACAAATATAAAACAATGAGTGAAGGCCCCGTCAA
	R:	TGACATAACTAATTACATGACTAATTATCCTTCGTATCTT
POS5	F:	TTACAACAAATATAAAACAATGTTTGTCAGGGTTAAATTT
	R:	TGACATAACTAATTACATGATTAATCATTATCAGTCTGTC
PNTB	F:	TTTACAACAAATATAAAACAATGTCTGGAGGATTAGTTAC
	R:	TGACATAACTAATTACATGATTACAGAGCTTTCAGGATTG

Table S2. Primers used for real-time quantitative PCR analysis

Genes	Primer sequences
IDP1	F: CTCCTGATGAAGCTCGTGTGAAGG
	R: CTGAACACTGTACCGCCGAGAATG
OYE2	F: CTGCTGGTGCCGATGGTGTTG
	R: TCGACAACTGCATCAACCACTTCC
ZWF1	F: GCCCTACTGGGTGACCATTC
	R: AATTTCCGGTGTTGGACCGT
POS5	F: AGATGGTACTCAGCTTCCGACGAC
	R: GTCATTCTCGGTCTTGGCGACAC
PNTB	F: TGGTGATTGGTGCTAACGATACGG
	R: ACCAGCATAGCCAGTGTTCATCG
GLR1	F: CTATTGCAGCGGGCAGAAAG
	R: AACCGGCTTCAGGATGTGAG
ALG9	F: CGCTATCTGTCCACTGGGTC
	R: TCGAATGCGGTTCTGATGGT

Table S3. Main metabolites yield (%) of the wild type and recombinant strains in the YPD medium without furfural

	Glycerol (%)	Acetic acid (%)	Ethanol (%)
BY	3.51	1.18	46.7
BY-POS5	4.60	1.05	44.8
BY-OYE2	3.94	1.00	47.3
BY-GLR1	3.97	1.28	48.3
BY-PNTB	4.91	0.73	45.0
BY-FDH	3.46	1.53	48.9
BY-IDP1	4.04	1.17	48.3
BY-ZWF1	3.70	1.22	49.3
BY-NDH	1.17	1.63	43.4
